# Supplementary material for: Hallucination filtering in radiology vision-language models using discrete semantic entropy
Source: Eur Radiol. 2026 Feb 20;36(7):6107–18. doi: 10.1007/s00330-026-12384-z (PMC13282299; doi:10.1007/s00330-026-12384-z)
Supplement: Supplementary file 1 — ELECTRONIC SUPPLEMENTARY MATERIAL [file 330_2026_12384_MOESM1_ESM.pdf]

Hallucination Filtering in Radiology Vision-Language Models Using Discrete Semantic Entropy

ELECTRONIC SUPPLEMENTARY MATERIAL

CLAIM: Checklist for Artificial Intelligence in Medical Imaging

Section / Topic	No.	Item	
**TITLE / ABSTRACT**			
	**1**	Identification as a study of AI methodology, specifying the category of technology used (e.g., deep learning)	**Vision-Language Model (GPT-4o; GTP-4.1)**
	**2**	Structured summary of study design, methods, results, and conclusions	**See abstract**
**INTRODUCTION**			
	**3**	Scientific and clinical background, including the intended use and clinical role of the AI approach	**The intended clinical role of DSE is safety-oriented selective answering/flagging of uncertain VLM outputs within radiology decision-support workflows (e.g., PACS integration) to reduce hallucination-related diagnostic risk; not intended for autonomous diagnosis.**
	**4**	Study objectives and hypotheses	**See end of Introduction (i–iii)**
**METHODS**			
Study Design	**5**	Prospective or retrospective study	**See methods (“retrospective”)**
	**6**	Study goal, such as model creation, exploratory study, feasibility study, non-inferiority trial	**Retrospective method-/evaluation study without model training; objective is to assess a black-box uncertainty metric (DSE) for VLM-VQA.**
Data	**7**	Data sources	**See methods (VQA-Med 2019; RadDataset per Huppertz et al.)**
	**8**	Eligibility criteria: how, where, and when potentially eligible participants or studies were identified (e.g., symptoms, results from previous tests, inclusion in registry, patient-care setting, location, dates)	**VQA-Med 2019: all 500 test image–question pairs included. RadDataset: all 206 de-identified 2D clinical cases as described by Huppertz et al.;no additional exclusions.**

	**9**	Data pre-processing steps	**No preprocessing of the public data**
	**10**	Selection of data subsets, if applicable	**Defined by previous publications**
	**11**	Definitions of data elements, with references to Common Data Elements	**Defined by previous publications**
	**12**	De-identification methods	**De-identification done by previous publications**
	**13**	How missing data were handled	**No missing data**
Ground Truth	**14**	Definition of ground truth reference standard, in sufficient detail to allow replication	**See methods (VQA-Med radiologist-validated answers; RadDataset diagnoses by four-radiologist consensus)**
	**15**	Rationale for choosing the reference standard (if alternatives exist)	**Defined by previous publications**
	**16**	Source of ground-truth annotations; qualifications and preparation of annotators	**Done by previous publications**
	**17**	Annotation tools	**Defined by previous publications**
	**18**	Measurement of inter- and intrarater variability; methods to mitigate variability and/or resolve discrepancies	**Defined by previous publications**
Data Partitions	**19**	Intended sample size and how it was determined	**Defined by previous publications**
	**20**	How data were assigned to partitions; specify proportions	**We didn’t train any models, so all data is testing data.**
	**21**	Level at which partitions are disjoint (e.g., image, study, patient, institution)	**We didn’t train the model, therefore not applicable**
Model	**22**	Detailed description of model, including inputs, outputs, all intermediate layers and connections	**GPT-4o and GPT-4.1 (see their model cards)**
	**23**	Software libraries, frameworks, and packages	**See https://github.com/TruhnLab/VisionSemanticEntropy**
	**24**	Initialization of model parameters (e.g., randomization, transfer learning)	**We didn’t train the models, therefore not applicable**
Training	**25**	Details of training approach, including data augmentation, hyperparameters, number of models trained	**We didn’t train the models, therefore not applicable**
	**26**	Method of selecting the final model	**We didn’t train the models, therefore not applicable**
	**27**	Ensembling techniques, if applicable	**N/A**
Evaluation	**28**	Metrics of model performance	**Accuracy, as classification was not binary and therefore**

			**sensitivity, specificity, AUROC, F1 and more are not applicable**
	**29**	Statistical measures of significance and uncertainty (e.g., confidence intervals)	**Confidence intervals and p-values (Table 1)**
	**30**	Robustness or sensitivity analysis	**See Table 1**
	**31**	Methods for explainability or interpretability (e.g., saliency maps), and how they were validated	**N/A**
	**32**	Validation or testing on external data	**We didn't train the models, therefore not applicable**
**RESULTS**			
Data	**33**	Flow of participants or cases, using a diagram to indicate inclusion and exclusion	**See Figure 1**
	**34**	Demographic and clinical characteristics of cases in each partition	**There was only test data which was described in previous publications.**
Model performance	**35**	Performance metrics for optimal model(s) on all data partitions	**See Results**
	**36**	Estimates of diagnostic accuracy and their precision (such as 95% confidence intervals)	**See Table 1**
	**37**	Failure analysis of incorrectly classified cases	**See Figure 3**
**DISCUSSION**			
	**38**	Study limitations, including potential bias, statistical uncertainty, and generalizability	**See 2nd last section of the Discussion**
	**39**	Implications for practice, including the intended use and/or clinical role	**See last section of the Discussion**
**OTHER INFORMATION**			
	**40**	Registration number and name of registry	**N/A**
	**41**	Where the full study protocol can be accessed	**The entire code to reproduce the results is available: https://github.com/TruhnLab/VisionSemanticEntropy**
	**42**	Sources of funding and other support; role of funders	**See Acknowledgments**
